# Deletions and duplications of the 22q11.2 region in spermatozoa from DiGeorge/velocardiofacial fathers

**DOI:** 10.1186/s13039-014-0086-3

**Published:** 2014-11-25

**Authors:** Laia Vergés, Òscar Molina, Esther Geán, Francesca Vidal, Joan Blanco

**Affiliations:** Unitat de Biologia Cellular (Facultat de Biociències). Universitat Autònoma de Barcelona, 08193-Bellaterra, Cerdanyola del Vallès, Spain; Current address: Wellcome Trust Center for Cell Biology, University of Edinburgh, Edinburgh, Scotland United Kingdom; Secció de Genètica Clínica. Hospital Universitari Sant Joan de Déu, 08950-Esplugues de Llobregat, Barcelona, Spain

**Keywords:** Deletions and duplications, DiGeorge/velocardiofacial syndrome, Non-allelic homologous recombination, Spermatozoa

## Abstract

**Background:**

DiGeorge/velocardiofacial syndrome (DGS/VCFS) is the most common deletion syndrome in humans. Low copy repeats flanking the 22q11.2 region confer a substrate for non-allelic homologous recombination (NAHR) events leading to rearrangements. This study sought to identify DGS/VCFS fathers with increased susceptibility to deletions and duplications at the 22q11.2 region in spermatozoa and to assess the particular contribution of intra-chromatid and/or inter-chromatid NAHR. Semen samples from nine DGS/VCFS fathers were analyzed by triple-color FISH using a probe combination that discriminated between normal, deleted and duplicated genotypes. Microsatellite analysis were performed in the parents and the affected children to determine the parental origin of the deleted chromosome 22.

**Results:**

A significant increase in 22q11.2 deletions was observed in the sperm of two out of nine DGS/VCFS fathers (odds ratio 2.03-fold, P < 0.01), and in both cases the deletion in the offspring was transmitted by the father. Patients with significant increases in sperm anomalies presented a disturbed deletion:duplication 1:1 ratio (P < 0.01).

**Conclusions:**

Altogether, results support that intra-chromatid NAHR is the mechanism responsible for the higher rate of sperm deletions, which is directly related to the transmission of the deleted chromosome 22 to offspring. Accordingly, the screening of sperm anomalies in the 22q11.2 region should be taken into account in the genetic counseling of DGS/VCFS families.

## Background

Diseases caused by chromosomal rearrangements because of architectural features of unstable regions are referred to as genomic disorders [1]. Regions involved in genomic disorders are flanked by low copy repeats (LCRs) [2] which, due to high degree of homology between their duplicated sequences (>95%), act as substrates for non-allelic homologous recombination (NAHR) [3,4].

**Figure 1:**
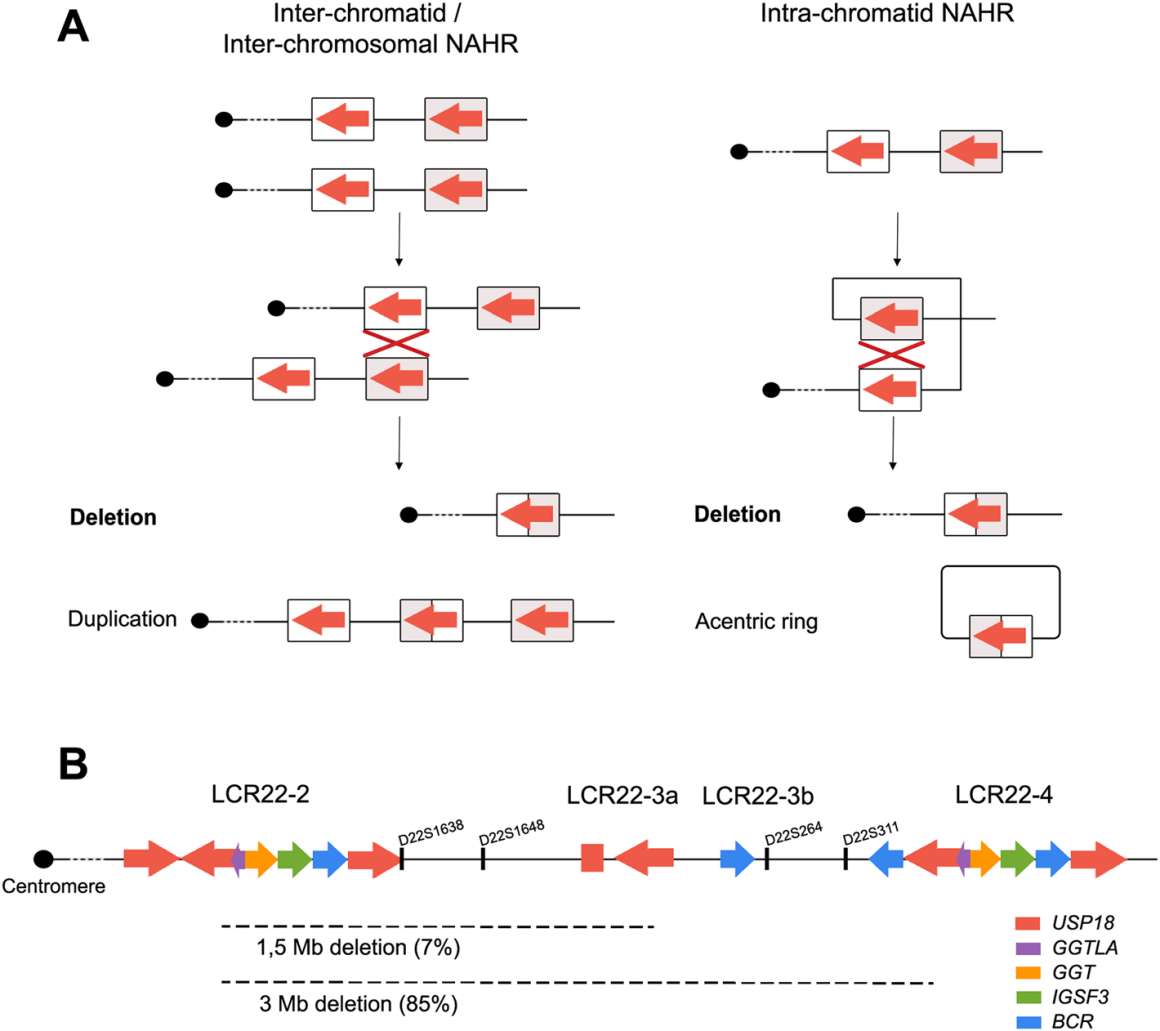
**Genomic rearrangements in the DGS critical region. A** Products of non-allelic homologous recombination (NAHR) between directly oriented LCRs. **B** Representation of 22q11.2 region and the most frequent DGS/VCFS deletions (adapted from Babcock et al. [6]). Colored arrows representative copies of the following genes and pseudogenes: USP18, GGTLA, GGT, IGSF3 and BCR.

Depending on the LCRs orientation and the number and type of chromatids involved, different rearrangements of the intervening segments are formed (Figure 1A): complementary deletions and duplications (inter-chromatid or inter-chromosomal NAHR between directly oriented LCRs), deletions and ring-acentric fragments (intra-chromatid NAHR between directly oriented LCRs) or inversions (intra-chromatid NAHR between indirectly oriented LCRs) [5].

The pericentromeric area of chromosome 22 contains eight distinct highly homologous LCRs comprising roughly 11% of the 22q11.2 region. According with their position in relation to the centromere, LCR22 regions are desingated as LCR22-2, LCR22-3a, LCR22-3b, LCR22-4, LCR22-5, LCR22-6, LCR22-7 and LCR22-8 [6]. It has been reported that these LCRs are involved in recurrent reorganizations causing different genomic disorders. Among them, DiGeorge/velocardiofacial syndrome (DGS/VCFS) (OMIM 188400/OMIM 192430) is the most common deletion syndrome in humans with an incidence of 1:4000 newborns [7]. The genetic cause is the haploinsufficiency of the genes comprised within the 22q11.2 region.

More than 85% of DGS/VCFS cases are caused by NAHR events involving LCR22-2 and LCR22-4 that give rise to a 3 Mb deletion [8]. In around 7% of cases, there are smaller deletions of 1.5 Mb flanked by LCR22-2 and LCR22-3a. The remaining cases are caused by unusual deletions involving distal LCR22s [9-11] (Figure 1B).

The duplication of the 22q11.2 region has been reported to cause a different phenotypic spectrum (22q11.2 duplication syndrome; OMIM 608363). It is important to mention that most of the duplications are the reciprocal products of 22q11.2 deletions originated by inter-chromatid or inter-chromosomal NAHR [12] (Figure 1A). Due to the milder phenotypes of duplications, which in some cases remain unnoticed, they have been probably underdiagnosed, thus compromising the estimation of their real incidence [13,14].

Concerning recurrence, the risk of DGS/VCFS and 22q11.2 duplication syndrome appears to be negligible, i.e. not different from the general population and has been established to be less than 0.5% [15]. The few familial cases described for DGS/VCFS have been related to a potential, unproven, parental mosaicism for a deletion in one of the progenitors [16,17]. Nevertheless, in several recurrent genomic disorders, some haplotypes have been suggested to predispose to NAHR events. In this sense, inversions of the critical regions have been reported in the fathers of children affected by deletion syndromes: Williams-Beuren [18-20], Prader-Willi [21], Angelman [22], Smith-Magenis [23] and 17q21.31 microdeletion [24], among several others [25]. In addition, copy number variations in the LCRs which flank critical regions have been described in fathers with affected offspring: Smith-Magenis syndrome [26], Williams-Beuren syndrome [27] or 16p12.1 microdeletion disease [28]. Moreover *trans* regulator factors of meiotic recombination, such as *PRDM9*, have also been associated with genomic disorders. In particular, certain *PRDM9* alleles have been suggested to predispose NAHR events of the regions 17p11.2 (Charcot-Marie-Tooth disease type 1A and hereditary neuropathy with liability to pressure palsies) [29], 7q11.2 (Williams-Beuren syndrome) [30] and 22q11.2 (DGS/VCFS) [31]. Finally, it has been described in the human germline an association between DNA hypomethylation of the critical regions and NAHR mediated by LCRs, suggesting the existence of an additional predisposing factor to NAHR based on methylation variations [32].

Thomas et al. [33] proposed a predominant role of inter-chromosomal NAHR in DGS cases, in agreement with previous observations by Edelmann et al. [34]. Similarly, previous results from our group in control donors showed no differences in the frequency of sperm 22q11.2 deletions and duplications, supporting inter-chromatid/chromosomal NAHR as the predominant mechanism for 22q11.2 rearrangements [35] (Figure 1A).

Fluorescence *in situ* hybridization (FISH) based methodologies in decondensed sperm nuclei allow the identification of gametes carrying deletions, duplications and inversions with a high sensitivity and specificity [21,35,36]. These interphase analyses, carried out in a large number of spermatozoa, offers the possibility of performing cell by cell analyses, thus assessing the incidence of rare events such as NAHR, and to establish a direct relationship between the genomic architecture and chromosomal instability during meiosis. Previous results from our group have pointed out an increased incidence of 15q11q13 deletions in spermatozoa from some Prader-Willi syndrome (PWS) fathers, suggesting a higher risk of transmission of deletions in these subjects [35].

The aim of the present study was to analyze the frequency of deletions and duplications occurring at the 22q11.2 region in spermatozoa of fathers with descendants affected by DGS/VCFS using sperm-FISH strategies. The analysis of the results will allow us to assess whether an increase in the susceptibility of generating deletions and duplications is present in these subjects and to assess the particular contribution of the intra-chromatid and/or inter-chromatid NAHR events in the generation of these anomalies.

## Results

Deletion and duplication frequencies in the 22q11.2 region were analyzed in a total of 90,776 sperm nuclei from nine fathers of DGS/VCFS individuals (Table 1). The mean frequency of deletions (±SEM) was 0.28% ± 0.05, ranging from 0.13 to 0.57% and the mean frequency of duplications (±SEM) was 0.11% ± 0.02, ranging from 0.05 to 0.20% (Table 1).

**Table 1:** **Sperm-FISH results in the DGS fathers**

**Case**	**Age**	**Normal**	**del 22q11.2**	**dup 22q11.2**	**del + dup**	**Others** ^**a**^	**Total**
DG-1	39	10000 (99.19)	24 (0.24)	11 (0.11)	35 (0.35)	47 (0.47)	10082
DG-2	48	10000 (98.40)	57 (0.57)*	**20 (0.20)**	77 (0.77)*	86 (0.86)	10163
DG-3	-	9927 (99.09)	19 (0.19)	13 (0.13)	32 (0.32)	59 (0.59)	10018
DG-4	43	10000 (99.30)	15 (0.15)	7 (0.07)	22 (0.22)	49 (0.49)	10071
DG-5	36	10096 (98.87)	52 (0.52)*	**12 (0.12)**	64 (0.64)*	51 (0.51)	10211
DG-6	30	10000 (99.24)	**28 (0.28)**	**5 (0.05)**	33 (0.33)	43 (0.43)	10076
DG-7	39	10000 (99.34)	18 (0.18)	7 (0.07)	25 (0.25)	41 (0.41)	10066
DG-8	33	9948 (99.31)	13 (0.13)	10 (0.10)	23 (0.23)	46 (0.46)	10017
DG-9	37	9930 (98.59)	25 (0.25)	16 (0.16)	41 (0.41)	31 (0.31)	10072
% ± SEM		99.04 ± 0.11	0.28 ± 0.05	0.11 ± 0.02	0.39 ± 0.06	0.50 ± 0.05	
% ± SEM^b^		99.08 ± 0.09	0.17 ± 0.02	0.12 ± 0.03	0.27 ± 0.05	0.64 ± 0.05	

^a^Disomies, diploidies and nullisomies.

^b^Control population data published in Molina et al., 2011 [36].

*Significant increases versus control data (P < 0.01).

Bold numbers indicate distortion of the 1:1 del:dup ratio (P < 0.01).

**Figure 2:**
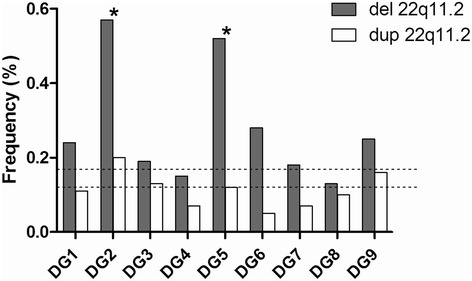
**Frequencies of 22q11.2 deletions and duplications observed in each DGS father.** Asterisks indicate cases with significant increases in deletions compared with control data (Molina et al. [36]). Dashed lines indicate deletion and duplication mean in controls (0.17 and 0.12, respectively).

No significant differences were observed in the frequency of deletions, duplications or del + dup between the population of DGS/VCFS fathers and our internal control data (Mann-Whitney test; P = 0.111, P = 1.000 and P = 0.191, respectively). At the individual level, we found significant increases in 22q11.2 deletions and del + dup in two out of nine DGS/VCFS fathers (Chi-square test; DG-2 and DG-5 P < 0.01) (Figure 2, Table 1). The risk to generate deletions of these individuals compared with the control population was estimated with an odds ratio of 2.03 (95% CI =1.63-2.54). On the other hand, there was no correlation between the frequencies of deletions, duplications or del + dup and the father’s age (Spearman correlation; P = 0.840, P = 0.360 and P = 0.752, respectively).

**Figure 3:**
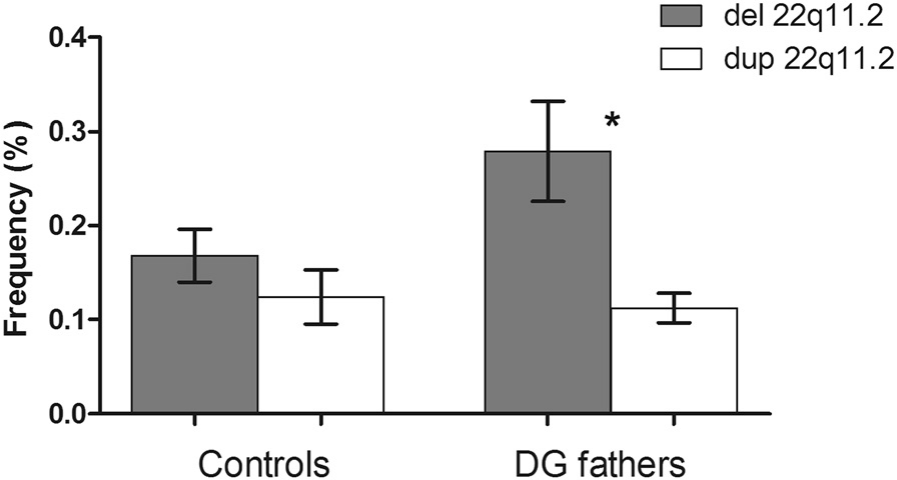
**Mean frequencies of 22q11.2 deletions and duplications in control donors (Molina et al. [** 36 **]) and DGS fathers.** Error bars represent the standard error of the mean (SEM) and asterisks indicate significant differences between the frequency of deletions and duplications.

Concerning the participation of the inter-chromatid and/or intra-chromatid NAHR in the generation of anomalies, no correlation was observed between deletion and duplication frequencies in DGS/VCFS fathers (Spearman correlation; P = 0.194). Furthermore, a significant increase in the mean frequency of 22q11.2 deletions was observed compared with the mean frequency of 22q11.2 duplications (Wilcoxon test; P = 0.004) (Figure 3). Individual comparisons showed significant increases in 22q11.2 deletions in three out of the nine cases analyzed (Chi-square test; DG-2, DG-5 and DG-6 P < 0.01) (Table 1); DG2 and DG5 correspond to individuals that showed significant increases in 22q11.2 deletions and del + dup compared to the control data.

The parental origin of the syndromes was ascertained in eight out of the nine DGS/VCFS families. In six out of the eight families, the origin of the deletion on chromosome 22 was paternal. In the remaining two cases, the origin was maternal (Table 2). Importantly, in the fathers that showed significant increases in 22q11.2 deletions and del + dup in spermatozoa, the origin of the deletion on chromosome 22 in the affected children was paternal.

**Table 2:** **Determination of the parental origin in eight DGS families: father (F), mother (M) and affected son (S)**

**CASES**	**D22S1638**	**D22S1648**	**D22S264**	**D22S311**	**D22S303**	**PARENTAL ORIGIN**
DG1F	114/116	174/174	185/195	**251/251**	220/222	PATERNAL
DG1M	116/116	174/174	195/195	**250/256**	222/223	
DG1S	116	174	195	**256**	220/223	
DG2F	116/116	170/172	**195/203**	**250/250**	212/222	PATERNAL
DG2M	116/116	172/172	**201/201**	**250/257**	212/222	
DG2S	116	172	**201**	**257**	222/222	
DG3F	**99/105**	174/178	193/199	**256/256**	220/223	PATERNAL
DG3M	**116/116**	174/174	193/199	**256/259**	222/224	
DG3S	**116**	174	193	**259**	220/224	
DG5F	116/120	174/174	**195/201**	250/256	224/225	PATERNAL
DG5M	116/118	174/174	**188/203**	256/256	224/226	
DG5S	-	174	**203**	256	224/226	
DG6F	**105/116**	**174/174**	**187/191**	250/250	222/222	MATERNAL
DG6M	**109/109**	**173/178**	**185/193**	250/254	222/222	
DG6S	**116**	**174**	**191**	250	222/222	
DG7F	**109/120**	174/174	185/199	**250/250**	222/224	PATERNAL
DG7M	**116/114**	174/174	199/205	**254/257**	212/224	
DG7S	**116**	174	199	**254**	212/224	
DG8F	**114/114**	174/174	187/199	252/256	213/223	PATERNAL
DG8M	**109/116**	174/174	199/201	250/256	223/225	
DG8S	**116**	174	199	256	223/223	
DG9F	**116/116**	**174/174**	**199/201**	**250/256**	212/222	MATERNAL
DG9M	**111/122**	**170/171**	**187/191**	**250/254**	222/222	
DG9S	**116**	**174**	**201**	**256**	212/222	

Informative microsatellites are indicated in bold.

## Discussion

The reliability of sperm-FISH analyses to detect deletions and duplications has already been demonstrated in previous studies [35,36]. The use of probes spanning the critical region, control probes for the chromosome involved and the application of strict scoring criteria allow the unequivocal identification of normal, deleted and duplicated genotypes.

In the population of DGS/VCFS fathers, no increased susceptibility in generating deletions and duplications was observed. Nevertheless, we found significant increases in sperm 22q11.2 deletions in two fathers who were among the ones who transmitted the deletion on chromosome 22 to their offspring. Focusing on cases with a paternally inherited deletion (n = 6; Table 2), two out of the six cases (33.3%) presented a higher risk for DGS/VCFS recurrence.

Similar situations have been observed in other studies where increases in chromosomal anomalies have been found in spermatozoa from transmitting fathers: 15q11q13 deletions in spermatozoa from Prader-Willi syndrome fathers [35], chromosome 21 disomies in spermatozoa from Down syndrome fathers [37], sex chromosomes disomies in spermatozoa from fathers with descendants affected by Turner syndrome [38] and Klinefelter syndrome [39]. Although the number of DGS/VCFS fathers studied in this work is limited and the anomaly increases observed are moderate, a comprehensive analysis of the literature suggest that increases in 22q11.2 anomalies could reach clinical relevance. Actually, the risk to generate deletions of these individuals was estimated to be twice the estimated risk in the control population (odds ratio 2.03-fold). Accordingly, these individuals should be considered at risk of transmitting DGS/VCFS to their descendants. We do believe that, in DGS/VCFS families with a 22q11.2 deletion transmitted by the father, the screening of sperm anomalies in the 22q11.2 region using sperm FISH should be taken into account to gather information that can help in the genetic reproductive counseling of families. The detection of higher rates of deletions in father spermatozoa should be an indication for a prenatal diagnosis.

Concerning the origin of the increased amount of deletions in these two fathers, although some authors have identified specific LCR architectures which promote genomic disorders [26-28], no predisposing haplotypes have yet been described for DGS/VCFS. Nevertheless, it is reasonable to think about the possible existence of a different LCR22s architecture in risky DGS/VCFS fathers, specifically affecting those LCRs mostly involved in the formation of deletions (LCR22-2 and LCR22-4). Baumer et al. suggested that variations in the number and position of LCRs involved in DGS/VCFS may result in higher rates of unequal crossing-over [40]. Another possible explanation comes from the influence of allelic variation at the *PRDM9* locus on the 22q11.2 mutation rate. In this regard, Alemany-Schmidt et al. have recently described a higher frequency of the *PRDM9* A allele in a small population of transmitting DGS/VCFS individuals suggesting a link between *PRDM9* allelic variation and NAHR events [31]. However, other authors have failed to identify this association [30]. Moreover, the description of *PRDM9* capacity for recognizing consensus sequences located within the LCRs, has also driven the suggestion that the activity of *PRDM9* depends on the number of binding motifs and the presence of specific SNPs within the LCR sequences [29,41].

In any case, further work needs to be done to establish the genetic factors that promote NAHR events in individuals with an increased risk of transmitting DGS/VCFS. This is an important task for the future that we have already embarked on, studying the LCR22s architecture using fiber-FISH strategies [42]. We envisage that this approach would allow the establishment of a direct relationship between specific LCR haplotypes and increased rates of NAHR in spermatozoa.

Regarding the NAHR mechanisms of the formation of reorganizations in the 22q11.2 region, the results obtained so far by our group have shown a predominant inter-chromatid NAHR in the 22q11.2 region of control donors [36] in agreement with other authors which established that most cases of DGS/VCFS are due to inter-chromosomal recombination [33,34]. In the present work, although a higher frequency of 22q11.2 deletions was detected in DGS/VCFS fathers compared with the frequency of duplications, the del:dup ratio in six out of nine DGS/VCFS fathers (66.7%) was equivalent to 1:1, reflecting major inter-chromatid NAHR events. In the remaining three DGS/VCFS fathers, we found a significant increase in deletions versus duplications and, hence, a preponderance of intra-chromatid NAHR. Importantly, two of them were classified as individuals at risk. In fact, we have described similar results in sperm-FISH studies in Prader-Willi syndrome fathers [35], i.e. a major inter-chromatid NAHR in control donors and a major intra-chromatid NAHR that causes higher rates of 15q11q13 deletions in risky fathers.

Many changes and structural rearrangements in the chromatin structure take place during pre-meiotic, meiotic, and post-meiotic stages of spermatogenesis. These rearrangements involve the formation of double-strand breaks (DSBs) which could be a source of genomic instability along spermatogenesis [43,44]. In this way, the intra-chromatid NAHR events may occur before meiosis what would give rise to germ cell mosaicism, thus increasing the chance of transmitting a deletion [40,45]. Indeed, germ line mosaicism has been proposed not only as the origin of genomic disorders [16,17,46,47], but also for other syndromes such as Down syndrome [48-50], suggesting that germ line mosaicism underlies the occurrence of many genetic diseases. Intra-chromatid NAHR arising during or after meiosis would be also likely to occur and would raise the deletion rates in sperm. Therefore, although the underlying mechanism remains elusive, it seems that the major genomic instability at LCR22s in risky DGS/VCFS fathers preferentially promote intra-chromatid NAHR events that could occur along all the entire spermatogenic process. In the presence of risky haplotypes, the formation of sperm deletions by other DSB repair systems, like non-homologous end joining (NHEJ) or fork stalling and template switching (FoSTeS) should be also considered [5].

## Conclusions

This study demonstrated an increased risk of transmitting DGS/VCFS in some men due to higher rates of deletions in spermatozoa. Although we are aware of the limitations of the sample size and that further work needs to be done, results deserve to be taken into consideration and the screening of 22q11.2 deletions in spermatozoa should be suggested in transmitting fathers of DGS/VCFS seeking reproductive genetic advice.

## Methods

### Biological samples

Semen samples were obtained from nine DGS/VCFS fathers aged between 30 and 48 years. All subjects were normozoospermic and showed normal karyotyes. Peripheral blood samples were collected from eight out of the nine DGS/VCFS families in EDTA-containing tubes (Family DG4 did not provide blood samples). Each family provided blood samples from the affected offspring and from the first-degree relatives (parents). To our knowledge, none of them had been exposed to genotoxic agents, and no history of chemotherapy, radiotherapy or chronic illness was recorded.

Parents gave their informed consent in writing to participate in the study and the protocols were approved by the Institutional Ethics Commitees of the two institutions collaborating in this study (Universitat Autònoma de Barcelona and Hospital Sant Joan de Déu, Barcelona, Spain).

### Fluorescence in situ hybridization on sperm

Semen samples were processed as described previously by our group; details of sperm fixation, nuclear decondensation and FISH protocol have been described elsewhere [51]. Briefly, the sperm fraction was resuspended in hypotonic solution (0.075 M KCl) for 30 minutes at 37°C and fixed in methanol:acetic acid (3:1) solution. Spermatozoa were spread on a slide and kept at -20°C. Prior to hybridization, sperm nuclei were decondensed by slide incubation at 37°C in Tris buffer containing 25 mmol/ml dithiothreitol and 1% Triton X-100 from 5 to 10 minutes.

**Figure 4:**
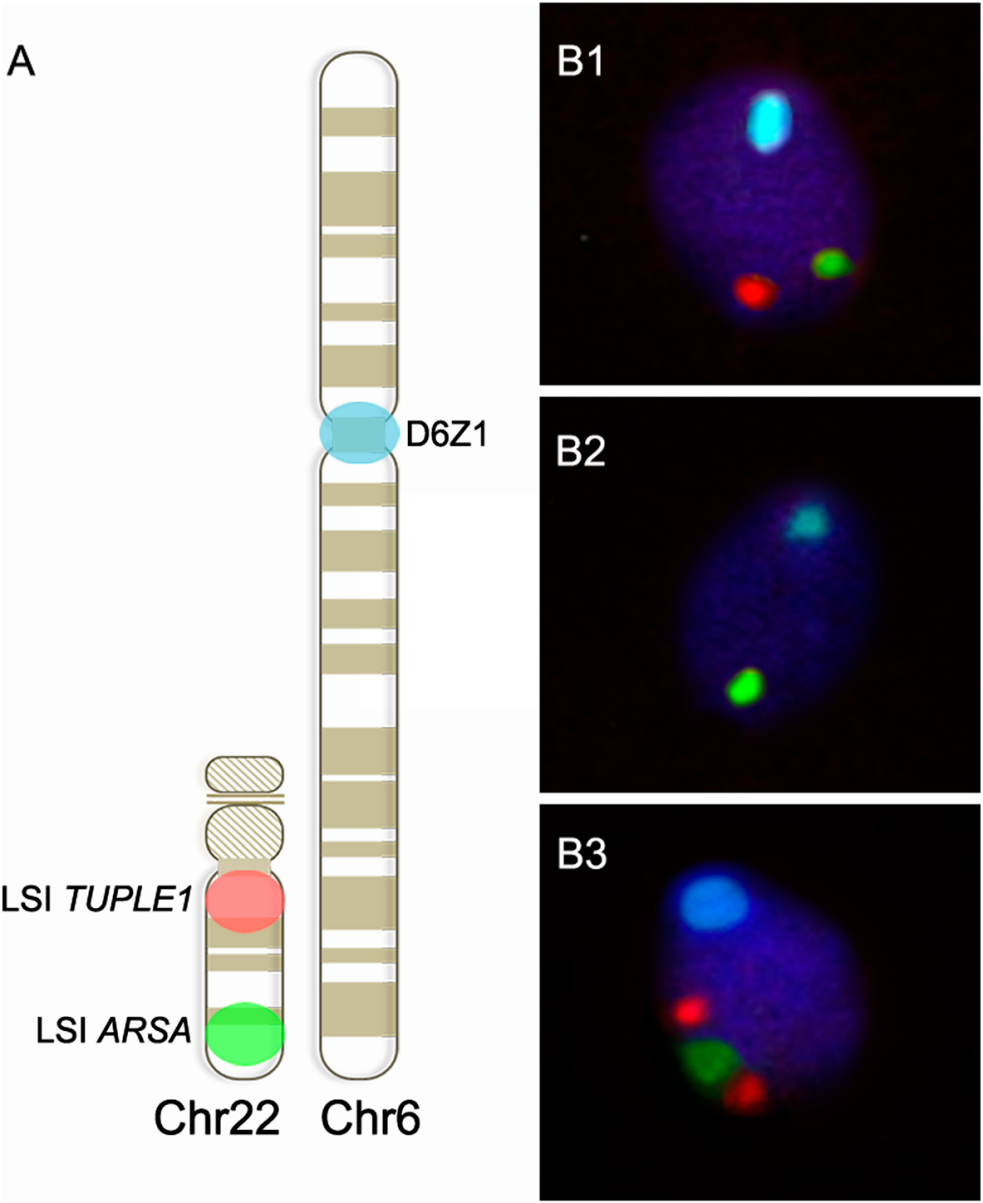
**FISH design. A**. Probe combination: LSI TUPLE1 at 22q11.2, Spectrum Orange; LSI ARSA at 22q13, Spectrum Green; and CEP6 at D6Z1 locus, Spectrum Aqua. B. Representative images of spermatozoa with normal **(B1)**, deleted **(B2)** and duplicated **(B3)** genotypes.

To determine the frequency of deletions and duplications in the critical 22q11.2 region, the following probe combination was used (Figure 4): 1) locus-specific (LSI) probe for *TUPLE1* localized inside the critical region at the 22q11.2 sub-band (*Spectrum Orange*; Abbott Molecular), 2) LSI probe for *ARSA* localized outside the critical region at the 22q13 sub-band (*Spectrum Green*; Abbott Molecular) and 3) centromeric probe recognizing the D6Z1 locus of chromosome 6 (CEP6, *Spectrum Aqua*; Abbot Molecular). LSI *ARSA* was used to discriminate between nullisomy/disomy 22 and 22q11.2 deletion/duplication genotypes, respectively, while the CEP6 probe was used as a ploidy control.

Analyses were carried out using an Olympus BX60 epifluorescence microscope equipped with a triple-band pass filter and specific filters for Aqua, FITC and Cy3. A minimum of 10,000 sperm nuclei were analyzed for every single father in two independent FISH experiments, applying strict assessment criteria [35].

### Statistical analysis

Data were analyzed using SPSS 15.0 software (SPSS Inc.; Chicago IL, USA) under the advice of the statistical service of the Universitat Autònoma de Barcelona. To avoid false positives due to the high number of spermatozoa analyzed per subject, differences were considered to be statistically significant when P < 0.01.

To assess the susceptibility in generating deletions and duplications, the mean frequency of 22q11.2 deletions, duplications and the sum of deletions and duplications (del + dup) of DGS/VCFS fathers were compared with the mean frequency observed in our own control datasets [36] using a Mann-Whitney test. Next, the frequencies of deletions, duplications and del + dup of every single father were compared with the basal frequencies observed in the control population using a Chi-square test. Additionally, Spearman correlations between the frequency of deletions, duplications and del + dup and the age of DGS/VCFS fathers were performed.

To assess the participation of inter-chromatid and/or intra-chromatid NAHR in the generation of anomalies, three comparisons were performed. A Spearman correlation test of 22q11.2 deletion and duplication frequencies was performed in DGS/VCFS fathers. In order to analyze whether the mean frequency of deletions was different from that of duplications, a Wilcoxon test and Chi-square test were performed at the population and individual level, respectively.

### Microsatellite analysis

To determine the parental origin of the deleted chromosome in the affected children, genomic DNA was isolated from 3 mL of peripheral blood samples collected in EDTA-containing tubes using the Gentra Puregene Blood kit (QUIAGEN Inc.) following the manufacturer’s instructions.

Five microsatellite markers distributed inside (D22S1638, D22S1648, D22S264, D22S311) and outside (D22S303) the deleted region were genotyped in every single DGS/VCFS family (Figure 1B; Table 3).

**Table 3:** **Microsatellite markers used to determine the parental origin**

**Microsatellite**	**Position**	**Primers**	**Labelling**	**Lenght (bp)**	**Annealing temperature**
**D22S1638** ^**a**^	22q11.21	F: GACAACAGCAAATTGCACATT	HEX	93	55°C
		R: TCACGCCACTACCCTCCAG			
**D22S1648** ^**a**^	22q11.21	F: CAGATGCTTCAGGAGAAGTG	HEX	152	50°C
		R: AGTTGTCAGATGCCTAAGAGA			
**D22S264** ^**a**^	22q11.21	F: ATTAACTCATAAAGGAGCCC	HEX	190-198	56°C
		R: CACCCCACCAGAGGTATTCC			
**D22S311** ^**a**^	22q11.21	F: GCTAGTGTGAGATAACGAAGCC	6-FAM	262	63°C
		R: TTTTTGTATTTTTAGTAGAGACGG			
**D22S303** ^**b**^	22q11.22	F: AGGACCTCAGACTGGTCAGTC	6-FAM	220-233	56°C
		R: CTCCCATGAGAAGGTACACTCC			

^a^Microsatellites inside the deleted region.

^b^Microsatellite outside the deleted region.

Table shows the forward (F) and reverse (R) primers, the 5′ labelling in the forward primers, the size of the amplified markers and their chromosomal position.

The microsatellite markers were amplified by PCR using dye-labeled primers (HEX or 6-FAM) and following standard procedures. PCR amplifications were performed in a reaction mixture containing 40-90 ng of genomic DNA, 25 pmol of each primer (Roche), 2.5 mM of each dNTP (Applied Biosystems), 25 mM MgCl_2_ (Applied Biosystems), 10x PCR buffer II (Applied Biosystems) and 1U AmpliTaq Gold DNA polymerase (Applied Biosystems). Amplifications were performed as follows: 94°C for 10 min; 35 cycles of 94°C for 30 s, an appropriate annealing temperature for 30 s, and 72°C for 35 s; and a final extension step of 72°C for 12 min.

Products were analyzed by capillary electrophoresis on a genetic analyzer (ABI3130 XL, Applied Biosystems) using Peak Scanner software, version 2.0 (Applied Biosystems).

Sperm FISH and microsatellite analyses were done in a blinded manner and the results were compared only at the end of the entire experiment.

## Abbreviations

FISH: Fluorescence in situ hybridization
NAHR: Nonallelic homologous recombination
LCRs: Low-copy repeats
DGS/VCFS: DiGeorge/velocardiofacial syndrome
